# Research and Remedies

**Published:** 1913-06-28

**Authors:** 


### RESEARCH AND REMEDIES.

### Subcutaneous Champagne.

The British Hospital in Buenos Ayres has had as senior medical officer for many years Dr. John O'Conor, a surgeon of much resource and high reputation. In the *Lancet* Dr. O'Conor invites attention to a method of treating surgical shock by means of subcutaneous injection of good dry champagne. The effects claimed are immediate and remarkable; the pulse gains volume, the skin becomes warm, the clammy sweat ceases, and within an hour tranquil sleep ensues. It is stated that this result exceeds in value that following saline infusions, and if that is so the method is certainly worth trial. A small bottle of champagne contains some 400 grammes (about 16 ozs.); this is emptied into a 500-gramme flask and the flask filled up with normal saline solution. The whole is then injected into the subcutaneous tissues, and repeated in six hours if necessary. No local trouble of any kind follows this procedure. Pituitary extract in combination with this treatment is strongly recommended. Dr. O'Conor does not offer any explanation of the mode of action of this stimulation, being content to record its actual existence and the fact that no after-depression follows it. Doubtless the "acapnia" school of physiologists will ascribe the benefits to the $CO_2$ contained in the champagne; though pituitrin, according to their theory, is positively harmful. The superiority of the best dry champagne over any other sort is also not easy to explain.

### Quinine and Cinchona.

The price of quinine salts is rising, due, it is said, to the operations of a syndicate which has got control of the chief sources of supply; and it is rumoured that further heavy rises are not improbable. Major Waters, of the Indian Medical Service, has experimented with two drugs, known as cinchona febrifuge and amorphous cinchona alkaloid, as substitutes for quinine in malarial cases. He finds that both these are much more powerful than the most efficacious of the quinine salts, so that proportionately smaller doses of them will cure the fever; and the cost is only about one-quarter of quinine. This conclusion is based upon a series of sixty-two consecutive cases of malaria treated with these drugs. They have, he reports in the *Indian Medical Gazette*, no unpleasant effects, can be given in small tablets, and take action very rapidly as well as effectually. It may be added that cinchona febrifuge is not a simple chemical substance, but a mixture of all the substances contained in cinchona bark in an amorphous form. The amorphous alkaloid is one of these constituents of the febrifuge, and its superior efficiency over quinine is said to be due to its ready assimilation by the system.

